# Whole genome sequencing and molecular characterization of two *Bacillus licheniformis* strains isolated from hot springs in yellowstone ational park

**DOI:** 10.3389/fbinf.2026.1867986

**Published:** 2026-07-08

**Authors:** Ola K. Elsakhawy, Mohamed A. Abouelkhair, Stephen A. Kania

**Affiliations:** 1 Department of Veterinary Biomedical Sciences, Rowan University, Glassboro, NJ, United States; 2 Department of Biomedical and Diagnostic Sciences, University of Tennessee, Knoxville, TN, United States

**Keywords:** antibiotic resistance, *Bacillus licheniformis*, bioinformatics, comparative genomics, pangenome analysis

## Abstract

*Bacillus licheniformis* is a Gram-positive, endospore-forming bacterium with broad biotechnological applications. Thermophilic environments such as hot springs may harbor strains with unique biosynthetic capabilities relevant to drug discovery. In this study, we isolated two *B. licheniformis strains* (S3 and S4) from the Five Sisters hot spring in Yellowstone National Park (68 °C and 65 °C, pH 8) and performed whole-genome sequencing using both the Oxford Nanopore long read and Illumina platforms. Hybrid *de novo* assembly using Unicycler yielded genome sizes of 4.80 Mbp (S3, 14 contigs) and 4.79 Mbp (S4, 22 contigs); GC contents were 45.12% and 45.10%, and N50 values were 4,546,802 bp and 2,415,736 bp, for S3 and S4, respectively. Both strains were assigned to Multi-Locus Sequence Typing sequence type ST-42. Pangenome comparison with 61 complete *B. licheniformis* genomes revealed an open pangenome of 10,374 genes, with 3,272 core genes, 430 soft core, 1,250 shell, and 5,422 cloud genes. AMRFinderPlus identified the *blaP*, encoding a class A beta-lactamase and its regulatory elements (*blaI* and *blaR1*); *erm(D)*, encoding a 23S rRNA methyltransferase conferring macrolide–lincosamide–streptogramin B resistance; and *catA*, encoding a chloramphenicol O-acetyltransferase that inactivates chloramphenicol through acetylation in both strains. A chromosomal *arsBC* locus was identified in both *B. licheniformis* S3 and S4, consistent with the arsenic-rich geothermal environment of Five Sisters hot spring. These findings highlight the biosynthetic potential of *B. licheniformis* strains isolated from extreme environments and provide a genomic foundation for future exploration of novel bioactive compounds with potential applications in drug discovery, agriculture, and biotechnology.

## Introduction

*Bacillus licheniformis* is a Gram-positive, endospore-forming, saprophytic, facultative anaerobic bacterium. It thrives in a wide variety of ecosystems, such as soil, plants, and oceans (Celandroni et al., 2019; Rey et al., 2004). *B. licheniformis* is taxonomically related to *Bacillus subtilis*, *Bacillus amyloliquefaciens*, and *B. paralicheniformis*, as indicated by comparisons of 16S rDNA and 16S-23S internal transcribed spacer (ITS) sequences (Dunlap et al., 2015; Xu and Côté, 2003). Several techniques are employed to distinguish *B. licheniformis* from *B. paralicheniformis*. These include 16S rRNA gene sequencing, matrix-assisted laser desorption ionization-time-of-flight (MALDI-TOF) analysis, and molecular techniques (Olajide et al., 2020). *B. licheniformis* has six unique housekeeping genes, which are *adk*, *ccpA*, *recF*, *rpoB*, *spo0A*, and *sucC* (Madslien et al., 2012).

Yellowstone National Park (YNP) thermal springs form a unique environment that harbors a wide variety of microorganisms (Podar et al., 2020). YNP geothermal environments present a variety of extreme conditions beyond just high temperatures. These conditions may include highly acidic or alkaline waters, as well as waters rich in sulfur. Bacteria growing in such environments have evolved strategies to thrive, including antibiotic production to inhibit other microorganisms and specialized metabolic pathways that enhance their survival under extreme conditions (Cavalier-Smith, 1992; Davies, 1994; Gunde-Cimerman et al., 2005).

*B. licheniformis* strains produce various antimicrobial compounds, including bacteriocin-like peptides, effective against Gram-positive bacteria and fungi (Nithya et al., 2012; Cai et al., 2019; He et al., 2006). Antimicrobial peptides produced by *B. licheniformis* DSM 13 are effective against a wide range of Gram-positive bacteria, including methicillin-resistant *Staphylococcus aureus* (MRSA) strains (Dischinger et al., 2009). Additionally, lichenin produced by *B. licheniformis* 26L-10/3RA inhibits the growth of *Streptococcus bovis* and *Eubacterium ruminantium* (Baruzzi et al., 2011). The surfactin produced by the fermentation of *B. licheniformis* exhibits significant antibacterial activity against *Clostridium perfringens* (Horng et al., 2019). *B. licheniformis* has been used in many biotechnological applications in agriculture, food, and pharmaceutical industries (de Boer et al., 1994; Li et al., 2020). Despite this recognized value, the genomic diversity and biosynthetic potential of *B. licheniformis* strains isolated from geothermal environments remain incompletely characterized.

In this study, we isolated two strains of *B. licheniformis* from hot springs in Yellowstone National Park and *de novo* assembled their genomes. We identified genes responsible for antibiotic resistance and biosynthetic gene clusters. The identification of these biosynthetic gene clusters will provide valuable insights into the potential antimicrobial properties of these strains. We believe that drug discovery can benefit significantly from studying organisms in harsh environments, as they may have developed specialized metabolic pathways to adapt to their extreme conditions (Marzban and Tesei, 2025; Safari et al., 2025). Overall, this study will enhance our understanding of antibiotic resistance and biosynthetic capacities in bacteria from remote environments.

## Materials and methods

### Sample collection and bacterial culture

We collected approximately 80 mL of sediment/water or biofilm/water from the YNP’s thermoalkaline Five Sisters hot springs (Permit YELL-2020-SCI-8152) (Peach et al., 2022). The hot springs are in an area restricted from public access and unlikely to be contaminated by human activity. Samples were collected in a sterile stainless-steel beaker mounted on a long pole. For S3, the water was 68 °C, and for S4 it was 65 °C. The pH was 8 for both samples. The samples were transferred to glass vials and sealed. They were stored at ambient temperature, transferred to a refrigerator, and shipped overnight to our laboratory at the University of Tennessee. Three types of liquid media were used for propagation: malt yeast, ATCC Medium 1,554 (mineral salt), and peptone-yeast-glucose. The samples were incubated aerobically and anaerobically for 10 days at 37 °C, 60 °C, and 70 °C. Broth cultures that grew were subcultured onto blood agar plates, and bacterial colonies were isolated. Preliminary taxonomic identification was performed by PCR amplification of the 16S rRNA gene followed by Sanger sequencing.

### Whole genome sequencing of bacterial isolates

Genomic DNA was purified using a Qiagen DNeasy UltraClean Microbial Kit (QIAGEN Inc.) following the manufacturer’s instructions. The quantity and quality of the genomic DNA were assessed using a NanoDrop 2000 spectrophotometer (Thermo Fisher Scientific, United States) and a Qubit fluorometer (Thermo Fisher Scientific, United States). Purified DNA was barcoded using the Native Barcoding Expansion 1–12 (EXP-NBD104), in conjunction with the Ligation Sequencing Kit (SQK-LSK109) (Oxford Nanopore, Oxford, United Kingdom) and further purified using AMPure XP beads (Beckman Coulter, United States). Sequencing, data collection, and base calling in high accuracy mode were performed using MinION flow cells (R9.4.1 FLO-MIN106, Oxford Nanopore), MinKNOW software v4.1.2, and Guppy basecaller v4.2.2 (ONT), respectively.

Illumina sequencing libraries were prepared using the tagmentation-based and PCR-based Illumina DNA Prep kits and custom IDT 10 bp unique dual indices (UDI) with a target insert size of 280 bp. No additional DNA fragmentation or size selection steps were performed. Illumina sequencing was performed on an Illumina NovaSeq 6,000 sequencer, producing 2 × 150 bp paired-end reads. Demultiplexing, quality control, and adapter trimming were performed with bcl-convert1 (v4.2.4).

### Genome assembly and MLST typing

Hybrid assemblies were performed using Unicycler version v0.5.0 (Wick et al., 2017), and assembled genomes were visualized using Proksee (Grant et al., 2023). Genome assembly statistics were computed using SeqKit2 (Shen et al., 2024). The genome sequence data were uploaded to the Type (Strain) Genome Server (TYGS), a free bioinformatics platform available under https://tygs.dsmz.de, for a whole genome-based taxonomic analysis (Meier-Kolthoff and Goker, 2019). The analysis also made use of recently introduced methodological updates and features (Freese et al., 2026; Meier-Kolthoff et al., 2022).

Pubmlst (https://pubmlst.org/organisms/bacillus-licheniformis) was used for Multi-Locus Sequence Typing (MLST) typing. The *Bacillus licheniformis* MLST scheme uses internal fragments of the following six housekeeping genes: *adk* (adenylate kinase), *ccpA* (transcriptional regulator), *recF* (recombination protein F), *sucC* (succinyl-CoA synthetase, subunit beta), *rpoB* (DNA-directed RNA polymerase, subunit beta), and *spo0A* (transcriptional regulator) (De Clerck and De Vos, 2004). Finally, BURST analysis was performed using all STs reported in pubmlst for *B. licheniformis* (https://pubmlst.org/bigsdb?db=pubmlst_blicheniformis_seqdef&page=plugin&name=BURST) (Jolley et al., 2018).

### Pangenome analysis

A total of 61 complete *B. licheniformis* genome assemblies were downloaded from NCBI RefSeq using the NCBI datasets command-line tool. Only assemblies at the “Complete Genome” assembly level were included according to NCBI assembly-level definitions, ensuring a consistent, high-quality comparative framework (O'Leary et al., 2024). These 61 references, together with the two study isolates, formed a dataset of 63 genomes for comparative analyses. Pairwise average nucleotide identity (ANI) was calculated for all 63 genomes using FastANI v1.34 in an all-versus-all comparison (Jain et al., 2018). Additionally, Mash v2.3 was used to compute pairwise distances among all genomes using a sketch size of 1,000 and a *k-mer* size of 21 (default parameters) (Ondov et al., 2016). The pangenome of the 63-genome dataset was characterized using Roary v3.13.0, which was run in Docker (staphb/roary:latest) with default parameters and a core gene definition threshold of 99% strain presence (Page et al., 2015). Gene categories were classified as core (99%–100% of strains), soft core (95–<99%), shell (15–<95%), and cloud (0–<15%). Core gene alignments generated by Roary were used to infer maximum-likelihood phylogenies with FastTree v2.2.0 double precision under the generalized time-reversible (GTR) model (Price et al., 2010). The genomic islands of both *B. licheniformis* S3 and S4 were predicted using Island Viewer 4 (Bertelli et al., 2017).

### Antimicrobial and arsenic resistance genes

To comprehensively screen for antimicrobial resistance (AMR) genes, genome assemblies were analyzed using AMRFinderPlus (v4.0.23, database v2025-07-16.1) in combined nucleotide-plus-protein mode (Feldgarden et al., 2021), with predicted protein sequences supplied from Prodigal v2.6.3 gene calls. Structural genes (*blaP*, *blaR1*) were accepted at default thresholds; the regulatory gene *blaI* was not returned by AMRFinderPlus and was therefore identified manually via open reading frame (ORF) extraction. The intergenic region between the *blaP* stop codon and the *blaR1* start codon (S3: 3,534,570–3,535,381; S4: 1,261,205–1,262,016; 811 bp in both strains) was translated in all six reading frames using Biopython v1.81. Each candidate ORF ≥60 aa was submitted to BLASTp (NCBI nr, restricted to *Bacillus*, April 2026) to assign function.

To identify arsenic resistance determinants, genome assemblies were screened using translated nucleotide BLAST (tBLASTn) with 52 curated arsenic resistance protein sequences from the AMRFinderPlus database (v2025-07-16.1) as queries. This approach enables the detection of divergent homologs without requiring prior gene calling. Searches were conducted with an *E*-value threshold of ≤1 × 10^−5^, minimum query coverage of 40%, and minimum amino acid identity of 30%. Hits were filtered and deduplicated by gene family per contig, retaining the highest-scoring alignment for each gene–contig pair. Chromosomal co-localization of resistance genes was assessed by comparing the genomic coordinates of high-confidence hits (*E*-value ≤1 × 10^−20^).

GC content of each resistance locus was computed over the exact annotated ORF boundaries and compared with the whole-genome mean (S3: 45.19%; S4: 45.10%) to assess horizontal gene transfer (HGT) signatures.

### Biosynthetic gene cluster detection and evaluation

Biosynthetic gene clusters (BGCs) were predicted using antiSMASH v7.1.0 executed via the official Docker image (antismash/standalone:7.1.0) on both *B. licheniformis* S3 and S4 genome assemblies (Blin et al., 2023). Ribosomally synthesized and post-translationally modified peptides (RiPPs) and (unmodified) bacteriocin were identified in *B. licheniformis* strains using the BAGEL4 webserver (http://bagel4.molgenrug.nl/). A comparison of biosynthetic gene clusters was conducted using Clinker (Gilchrist and Chooi, 2021).

## Results

### Assembly quality and genome completeness

**FIGURE 1:**
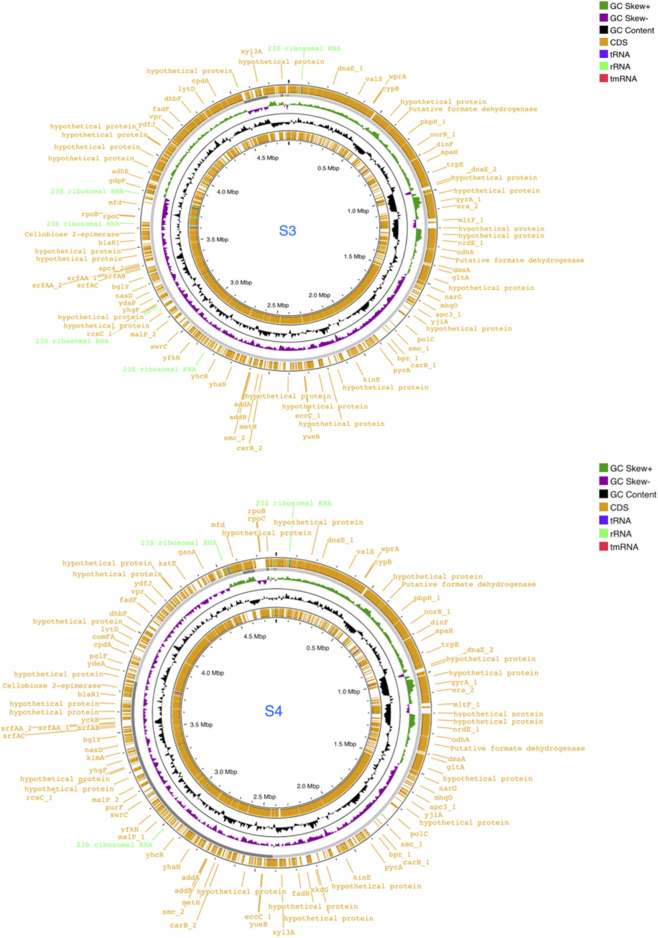
Circular genome map representing the genomes of *B. licheniformis* S3 and *B. licheniformis* S4 isolates. Marked information is displayed from the inner circle to the outermost, as follows: genome size, GC content, GC skew (+/−), and *B. licheniformis* gene annotations.

Genome assembly statistics computed using SeqKit v2 are presented in Table 1. The *B. licheniformis* S3 genome assembly has 14 contigs, a GC content of 45.12%, 5,141 CDSs, 24 rRNAs, 1 tmRNA, and 89 tRNAs (Figure 1). The *B. licheniformis* S4 genome assembly contains 22 contigs, 45.10% GC, 5,145 CDSs, 17 rRNAs, 1 tmRNA, and 89 tRNAs (Figure 1).

**TABLE 1:** Genome assembly and sequencing statistics for *Bacillus licheniformis* strains S3 and S4.

Metric	*Bacillus licheniformis* S3	*Bacillus licheniformis* S4
Total sequencing bases (bp)	1,291,383,600	1,300,488,600
Total raw reads (R1 + R2)	8,609,224	8,669,924
Read length (bp)	150	150
Number of contigs	14	22
Total assembly length (bp)	4,801,673	4,794,362
Sequencing coverage (×)	∼269×	∼271×
GC content (%)	45.12	45.10
N50 (bp)	4,546,802	2,415,736

**FIGURE 2:**
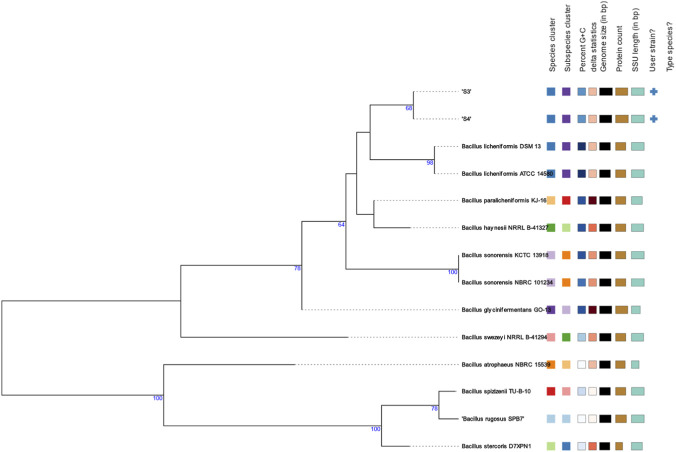
Tree inferred with FastME 2.1.6.1 from GBDP distances calculated from 16S rDNA gene sequences. The branch lengths are scaled in terms of GBDP distance formula *d*_*5*_. The numbers above branches are GBDP pseudo-bootstrap support values >60% from 100 replications, with an average branch support of 71.3%. The tree was rooted at the midpoint (Lefort et al., 2015; Farris, 1972).

The TYGS program, used to construct a 16S rDNA phylogenetic tree using the Genome BLAST Distance Phylogeny (GBDP) approach, identified *B. licheniformis* DSM 13 (ATCC 14580) as the most similar strain (Figure 2 **)**.

**FIGURE 3:**
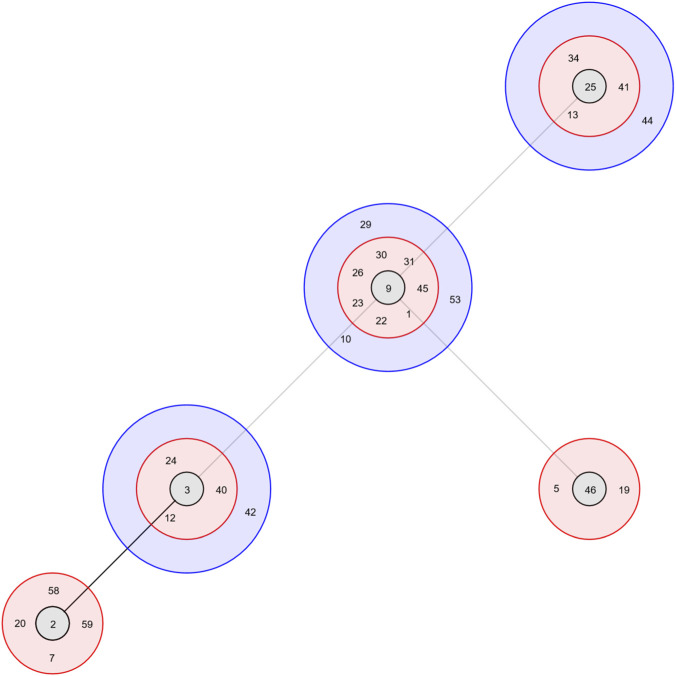
Analysis of clonal complexes of *B. licheniformis* S3 and *B. licheniformis* S4 isolates using the eBURST algorithm. Each node represents a distinct sequence type (ST), and lines indicate relationships based on allelic differences among the housekeeping genes of the MLST scheme.

Both *B. licheniformis* S3 and *B. licheniformis* S4 were assigned to ST-42 Table 2. Population structure and clonal relationships among typed isolates were examined using the eBURST (Based Upon Related Sequence Types) algorithm. Single-locus variants (SLVs), defined as isolates differing at exactly one of the six MLST loci, were used to construct a minimum spanning tree of clonal relationships. Clonal complexes (CCs) were defined as groups of three or more related STs connected through SLV relationships, with the most common ST designated as the founder of each complex (Figure 3).

**TABLE 2:** *B. licheniformis* S3 and *B. licheniformis* S4 MLST typing.

Strains	House-keeping genes	Allele	Length	Contig	Position (start- end)
*B. licheniformis* S3	*adk*	2	465	1	3640533–3640997
	*ccpA*	3	561	1	175427–175987
	*recF*	1	561	1	3786346–3786906
	*rpoB*	1	495	1	3663574–3664068
	*spo0A*	3	558	1	745432–745989
	*sucC*	2	549	1	1792387–1792935
*B. licheniformis* S4	*adk*	2	465	5	13697–14161
	*ccpA*	3	561	1	175466–176026
	*recF*	1	561	3	759897–760457
	*rpoB*	1	495	5	36738–37232
	*spo0A*	3	558	1	745471–746028
	*sucC*	2	549	1	1792426–1792974

### Pangenome analysis

**FIGURE 4:**
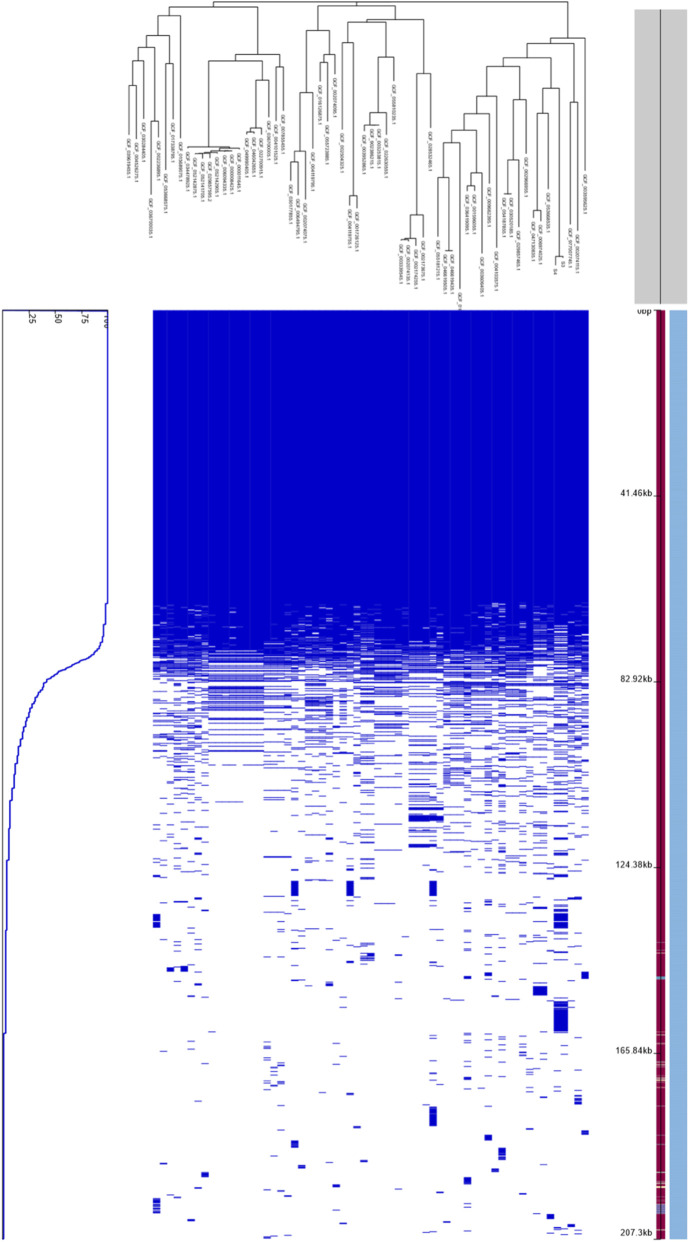
Pangenome analysis of *B. licheniformis* S3 and *B. licheniformis* S4 genomes using Roary. The left panel shows a maximum-likelihood phylogenetic tree constructed from single-nucleotide polymorphisms (SNPs) in the core genome. The right panel presents a Roary gene presence/absence matrix. Blue shading indicates the presence of a gene, while white denotes its absence.

The pangenome of the 63-genome dataset revealed an open pangenome architecture for *B. licheniformis*. Roary identified a total pan-genome of 10,374 genes, of which 3,272 (31.6%) were core genes, 430 (4.1%) soft core, 1,250 (12.0%) shell, and 5,422 (52.3%) cloud genes. Phylogenomic trees were reconstructed from core-gene alignments using FastTree under the GTR model, and UPGMA dendrograms were generated from ANI and Mash distances **(** Figure 4 **)**.

**FIGURE 5:**
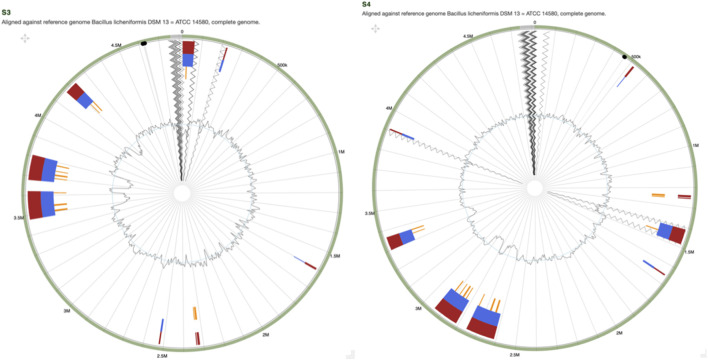
*B. licheniformis* Strains S3 and S4 aligned against reference genome *Bacillus licheniformis* DSM 13 = ATCC 14580 to predict the genomic islands. The green outer circle represents the scale line of the genome in Mbps, and the obtained genomic islands are represented by the different colors depending on the method as the following: Island Path-DIMOB (blue), SIGI-HMM (orange), and integrated detection (red). The inner circle represents the CG content, gray lines indicate contig boundaries.

Genomic islands of *B. licheniformis* S3 and S4 were predicted using IslandViewer 4, with *B. licheniformis* DSM 13 serving as the reference genome (Bertelli et al., 2017). Both strains possessed nine genomic islands each (Figure 5), encoding 735 and 751 genes in S3 and S4, respectively. Island sizes ranged from 4,128 to 144,585 bp.

### Antimicrobial resistance determinants

AMRFinderPlus detected the class A beta-lactamase *blaP* (EXACTP match, 100% amino-acid identity) and the sensor histidine kinase *blaR1* (HMM match, 33% identity) in *B. licheniformis* S3 and S4. The genes are arranged in tandem on the chromosome, separated by an 811-bp intergenic region that is identical in sequence between *B. licheniformis* S3 and S4. Canonical *Bacillus* beta-lactam resistance clusters encode a three-component inducible system — the serine beta-lactamase *BlaP*, the signal-transducing sensor kinase *BlaR1*, and the transcriptional repressor *BlaI* — in the order *blaP–blaI–blaR1* (Filée et al., 2003; Golemi-Kotra et al., 2003).

Manual ORF analysis of the *blaP–blaR1* intergenic interval identified a 387-bp open reading frame encoding a 129-aa protein in *B. licheniformis* S3 and S4. BLASTp returned the top hit as a BlaI/MecI/CopY-family transcriptional regulator from *Bacillus licheniformis* (WP_216911806.1, 100% identity, *E* = 2.2 × 10^−89^) with an identical match to APJ25573.1 from *Bacillus* sp. H15-1 (100% identity, 130/130 aa, *E* = 5.6 × 10^−91^). The *blaI* protein sequence is identical between *B. licheniformis* S3 and S4, confirming that the complete *blaP–blaI–blaR1* is present in *B. licheniformis* S3 and S4 Table 3.

**TABLE 3:** Genomic coordinates and GC content of the *blaP*–*blaI*–*blaR1* inducible beta-lactam resistance in *B. licheniformis* S3 and S4.

Gene	S3 coordinates	S4 coordinates	Size (bp)	GC% (S3/S4)
*blaP*	3,533,647–3,534,570	1,260,282–1,261,205	924	44.0%/44.0%
*blaI*	3,534,992–3,535,379	1,261,618–1,262,014	387	35.6%/35.9%
*blaR1*	3,535,381–3,537,180	1,262,016–1,263,815	1,800	39.7%/39.7%

GC content analysis revealed that *blaP*, the structural effector gene, has a GC content of 44.0% in both strains, while the regulatory genes *blaI* and *blaR1* are strongly AT-biased Table 3.

Additionally, *erm(D)* was identified in both isolates, which encodes a 23S rRNA (adenine-2058-N6)-methyltransferase (94.41% identity to WP_003183781.1, 99.65% coverage), conferring resistance to macrolide, lincosamide, and streptogramin B antibiotics.

A gene encoding a type A chloramphenicol O-acetyltransferase (*catA*) was identified in both isolates by profile HMM search (HMM accession NF000491.1; reference protein WP_087344902.1). The predicted CatA proteins showed 56% amino acid identity to the reference sequence across 98.6% of its length (216/217 residues aligned), consistent with a divergent but functional homolog. CatA enzymes confer chloramphenicol resistance by O-acetylating the antibiotic, preventing it from binding its ribosomal target.

The tBLASTn screening identified a chromosomal arsenic resistance locus in both isolates Table 4. The core of this locus consists of *arsB*, encoding an arsenite efflux membrane pump responsible for As(III) export, and *arsC*, encoding a thioredoxin-dependent arsenate reductase that catalyzes the initial detoxification step of As(V) reduction to As(III). In both isolates, *arsB* and *arsC* were co-localized within a ∼500 bp window separated by an intergenic gap of 77 bp, consistent with a canonical chromosomal *ars* resistance operon. In S3 and S4, this locus maps to contig 1 at positions 1,458,387–1,460,115 and 1,458,426–1,460,115, respectively. Both arsB and arsC showed high-identity matches (71% and 87% amino acid identity, respectively), with *E*-values of ≤8.5 × 10^−173^ and ≤1.6 × 10^−83^. In addition, both isolates harbored a high-confidence match with *arsN1a* (arsinothricin N-acetyltransferase, ArsN1 family A; 68% identity, *E*-value ≤1.3 × 10^−71^) at a distinct chromosomal position.

**TABLE 4:** Arsenic resistance genes identified in *B. licheniformis* S3 and S4 with corresponding amino acid sequence identity (%) to reference proteins.

Gene	Encoded protein/function	*B. licheniformis* S3	*B. licheniformis* S4
*arsB*	Arsenite efflux membrane pump	**71%**	**71%**
*arsC*	Arsenate reductase	**87%**	**87%**
*arsN1a*	Arsinothricin N-acetyltransferase ArsN1a	**68%**	**68%**

Bold values denote the amino acid sequence identities of arsenic resistance genes identified in both *B. licheniformis* S3 and S4.

### Biosynthetic gene cluster detection

AntiSMASH analyses revealed that *B. licheniformis* S3 and S4 harbor ten BGCs Table 5. BGC types identified included representatives of non-ribosomal peptide synthetase (NRPS) pathways, ribosomally synthesized and post-translationally modified peptides (RiPPs), polyketide synthases (PKS), terpenoid biosynthesis, and siderophore assembly. Three BGCs showed 100% KnownClusterBlast similarity to entries in the MIBiG database. Region 6 matched the lichenysin A biosynthetic cluster (BGC0000381; 100% similarity, 16/16 hits), consistent with the hallmark lipopeptide biosurfactant produced by *B. licheniformis* (Anuradha S, 2010; Yeak et al., 2022).

**TABLE 5:** BGCs predicted by antiSMASH in *B. licheniformis* S3 and S4 genomes.

BGC No.	Predicted type	Size (kb)	Best MIBiG match	Known compound	Similarity (%)	Biosynthetic category
1	T3PKS	41.1	—	Unknown polyketide	—	Polyketide (T3PKS)
2	Terpene	21.9	—	Unknown terpenoid	—	Terpene
3	Betalactone	28.5	BGC0001095	Fengycin (partial)	53	NRPS/PKS
4	NI-siderophore	33.5	BGC0002683	Schizokinen	60	Siderophore (hydroxamate)
5	Thiopeptide + LAP	41.3	BGC0000693	Butirosin A/B (weak)	7	RiPP (thiopeptide/LAP)
6	NRPS (lichenysin)	65.4	BGC0000381	Lichenysin A	100	NRPS lipopeptide
7	Lanthipeptide class II	27.0	BGC0000525	Lichenicidin VK21	100	RiPP (lanthipeptide)
8	RRE-containing lassopeptide	22.7	—	Novel lasso peptide	—	RiPP (lassopeptide)
9	NRP-metallophore + NRPS	51.7	BGC0002695	Bacillibactin	100	Siderophore (catecholate)
10	CDPS	20.8	BGC0002103	Pulcherriminic acid	66	Cyclodipeptide

Region 7 showed 100% similarity to the lichenicidin VK21 cluster (BGC0000525; 3/3 hits), a class-II lanthipeptide bacteriocin previously characterized in *B. licheniformis*. Region 9, annotated as an NRP-metallophore and NRPS cluster, matched the bacillibactin siderophore cluster (BGC0002695; 100% similarity, 10/10 hits). Bacillibactin is a catecholate-type siderophore responsible for high-affinity ferric iron (Fe^3+^) acquisition under iron-limiting conditions. It is crucial for bacterial growth and survival (Zhou et al., 2018). Previous studies have shown that bacillibactin has antibacterial activity (Nalli et al., 2022; Chakraborty et al., 2022). Region 4, classified as an NI-siderophore cluster, matched the schizokinen hydroxamate siderophore cluster (BGC0002683; 60% similarity, 6/10 hits), indicating the presence of a second, hydroxamate-type iron acquisition system in addition to the catecholate bacillibactin cluster. Region 10, annotated as a cyclodipeptide synthase (CDPS) cluster, matched the pulcherriminic acid pathway (BGC0002103; 66% similarity, 4/6 hits). Pulcherriminic acid serves as a precursor to pulcherrimin, an iron-chelating yellow pigment with antimicrobial properties. Region 3, predicted as a betalactone cluster, showed 53% similarity to the fengycin lipopeptide cluster (BGC0001095; 8/15 hits). Although fengycin biosynthesis is canonical in *B. subtilis*, the partial similarity and distinct cluster architecture suggest that region 3 may encode a structurally related but divergent lipopeptide or polyketide compound. Fengycin is a cyclic lipopeptide antibiotic produced by numerous *Bacillus* species, recognized for its strong antifungal, antibacterial, and biosurfactant properties (Zeng et al., 2021; Steller et al., 1999; Vanittanakom et al., 1986).

Four BGCs lacked a significant KnownClusterBlast match. Region 1 was predicted as a type III polyketide synthase (T3PKS) cluster. Region 8 (22.7 kb; 4,128,484–4,151,218 bp) was predicted to encode an RRE-containing lassopeptide. Region 2 was classified as a terpene biosynthetic cluster. Region 5 was annotated as a thiopeptide/linear azole-containing peptide (LAP) cluster.

**FIGURE 6:**
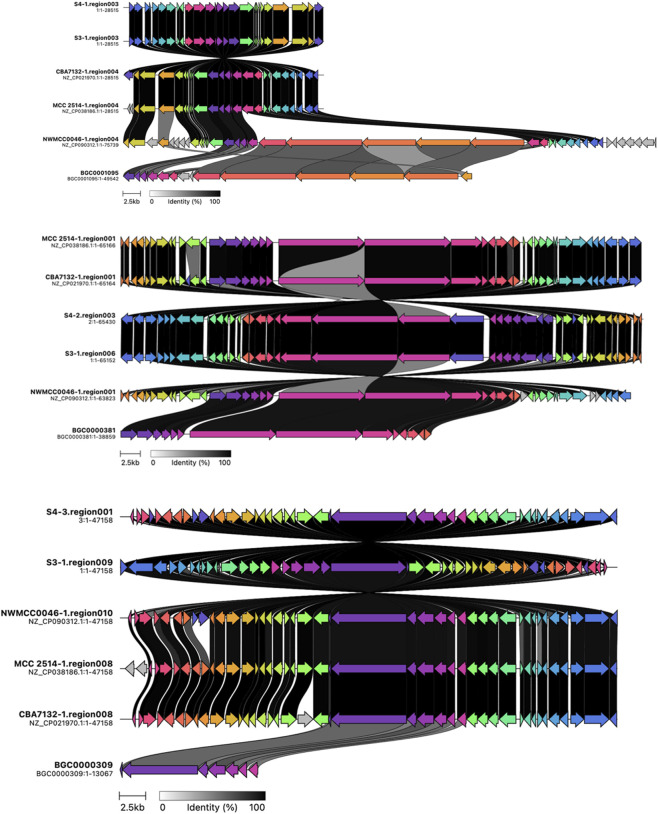
Comparison of fengycin, lichenysin, and bacillibactin regions across other *B. licheniformis* strains and reference MIBiG using Clinker.

Three *B. licheniformis* strains were included for comparative biosynthetic gene cluster analysis: MCC 2514 (NZ_CP038186) from raw milk, India; NWMCC0046 (NZ_CP090312) from soil, China; and CBA7132 (NZ_CP021970) from human feces, South Korea (Figure 6). The predicted BGCs appeared distinct from these non-thermophilic strains and known reference clusters, although experimental validation is required to determine whether they are functional and associated with active metabolite production.

To evaluate whether any BGC identified by antiSMASH v7.1.0 showed evidence of horizontal acquisition, GC content was computed for each complete BGC region independently in *B. licheniformis* S3 and S4 using Biopython v1.81. Region boundaries were taken directly from antiSMASH region-level feature annotations in the JSON output. GC content analysis of the ten BGC regions identified in *B. licheniformis* S3 and S4 revealed marked heterogeneity, ranging from 43.72% (T3PKS, Region 1) to 49.07% (NI-siderophore, Region 4), relative to a genome-wide mean of 45.1%.

To independently validate antiSMASH RiPP predictions and screen for bacteriocin clusters not detected by antiSMASH, the *B. licheniformis* S3 and S4 genome assemblies were submitted to the BAGEL4 web server. BAGEL4 returned five areas of interest (AOIs) for *B. licheniformis* S3 and six AOIs for *B. licheniformis* S4 (Table 6 **)**. The lichenicidin lanthipeptide cluster was confirmed by BAGEL4 as LichenicidinVK21A1, matching the 100% similarity score for BGC0000525 (lichenicidin VK21) in antiSMASH. Additionally, the lasso peptide cluster was confirmed, consistent with antiSMASH Region 8 (RRE-containing/lassopeptide). BAGEL4 resolves a 41.3kb thiopeptide/LAP region, identified by AntiSMASH, into two overlapping sub-clusters: Bottromycin AOI and Sonorensin AOI. BAGEL4 identified a competence peptide that antiSMASH did not detect. ComX/CSPs are quorum-sensing pheromones that initiate natural competence for DNA uptake and have been documented in *Bacillus subtilis* and related species (Aspiras et al., 2004). BAGEL4 identified a sactipeptide AOI in *B. licheniformis* S4. Sactipeptides are ribosomally synthesized peptides with antimicrobial activity against Gram-positive bacteria (Ruiz-De-Anda et al., 2025).

**TABLE 6:** BAGEL4 Areas of Interest (AOIs) identified in *B. licheniformis* S3 and S4 genomes.

*B. licheniformis* Isolate	BAGEL4 Area of Interest (AOI)	Start	End	GC%	BAGEL4 class
S3	AOI_01	4,038,671	4,063,486	46.32%	LichenicidinVK21A1
S3	AOI_02	4,128,824	4,148,824	45.02%	Lasso peptide
S3	AOI_03	2,689,136	2,709,136	47.44%	Bottromycin
S3	AOI_04	2,705,237	2,725,540	47.68%	Sonorensin
S3	AOI_05	2,295,574	2,315,691	44.58%	Competence peptide
S4	AOI_01	431,873	452,176	47.68%	Sonorensin
S4	AOI_02	22,209	42,326	44.58%	Competence peptide
S4	AOI_03	415,772	435,772	47.44%	Bottromycin
S4	AOI_04	483,314	508,129	46.32%	LichenicidinVK21A1
S4	AOI_05	397,976	417,976	45.01%	Lasso peptide
S4	AOI_06	277,154	297,154	47.16%	Sactipeptide

## Discussion

In this study, we isolated, sequenced, and *de novo* assembled the entire genomes of two *B. licheniformis* strains from hot springs in Yellowstone National Park. Then we performed pangenome analysis of the assembled genomes of *B. licheniformis* S3 and *B. licheniformis* S4, in the context of 61 publicly available complete genomes. The pangenome analysis revealed that approximately one-third of the genes are shared across the species (core genome), while over half belong to the accessory genome, reflecting the extensive genomic plasticity of *B. licheniformis*. This open pangenome structure aligns with previous reports and is consistent with the broad ecological distribution and metabolic versatility of the species. Pan-genomes encompass all genes across all *B. licheniformis* strains, comprising core and accessory genomes. The core genome comprises all genes shared by all *B. licheniformis* strains, whereas the accessory genome comprises both the cloud and shell genomes (Zhou et al., 2020; Inglin et al., 2018). Core genes are the housekeeping genes of bacteria, encoding proteins that are involved in essential biological activities such as DNA replication, transcription, translation, energy metabolism, and cell division. Accessory genes play a role in the evolution and adaptation of bacteria to various environmental conditions. Accessory genes encode proteins responsible for antibiotic resistance, virulence factors, specialized metabolic pathways, and the ability to utilize specific nutrients. Bacteria can acquire or expand their accessory genes through horizontal gene transfer, with new genes arising from non-coding or intergenic regions of the genome, gene duplication, and divergence (Daubin and Ochman, 2004; Abby et al., 2012; Subedi et al., 2019; Croucher et al., 2014).

Antimicrobial resistance genes were characterized in *B. licheniformis* strains. The *ermD* gene has been identified in thermotolerant *B. licheniformis* strains and a soil isolate (Ngom et al., 2023; Agersø et al., 2019; Jeong et al., 2020). The *ermD* gene encodes the erythromycin resistance methylase, which modifies the 23S rRNA component of the bacterial ribosome, conferring resistance to macrolides, lincosamides, and streptogramin B antibiotics (MLS) (Gryczan et al., 1984). These antibiotics are used to treat a variety of bacterial infections, including respiratory tract infections, skin infections, and sexually transmitted diseases. The *ermD* gene is a member of a larger family of genes known as the erm gene family (erythromycin ribosome methylation genes). Furthermore, the *blaP* gene was found in both *B. licheniformis* S3 and *B. licheniformis* S4. The *blaP* gene encodes a class A beta-lactamase, which confers resistance to beta-lactam antibiotics. The *in silico* identification of genes associated with antibiotic resistance is a critical step in understanding the mechanisms of resistance; however, it is essential to recognize that this identification alone does not guarantee the presence of antibiotic resistance. Experimental validation is necessary to establish a direct correlation between the identified genes and their functional role in conferring resistance. Their expression and functionality can be influenced by various factors, including environmental conditions and the presence of other regulatory elements. Additionally, the resistance profile of *B. licheniformis* strains may vary, and other resistance mechanisms can also contribute to antibiotic resistance.

The identification of a chromosomal *arsBC* locus in *B. licheniformis* S3 and S4 isolates is consistent with the geochemical characteristics of their environment. Five Sisters Springs is a geothermal spring system characterized by elevated dissolved arsenic concentrations, a condition that imposes strong selective pressure on resident microbial communities to acquire or maintain inorganic arsenic detoxification mechanisms. The *ars* operon — minimally comprising *arsC*, which reduces less mobile pentavalent arsenate As(V) to trivalent arsenite As(III), and *arsB*, which exports As(III) across the cytoplasmic membrane — represents the primary chromosomal arsenic resistance strategy in Gram-positive bacteria. Additionally, *B. licheniformis* S3 and S4 possess *arsN1a*, a resistance acetyltransferase that inactivates arsinothricin, a naturally produced organoarsenic antibiotic.

The antiSMASH analysis revealed a diverse and functionally rich secondary metabolite repertoire in both *B. licheniformis* S3 and S4, encompassing ten BGC types spanning NRPS lipopeptides, lanthipeptide bacteriocins, siderophores, polyketides, terpenoids, and other RiPP classes. This breadth is consistent with *B. licheniformis* being recognized as a prolific secondary metabolite producer and a commercially exploited source of bioactive compounds (Dischinger et al., 2009). Three distinct clusters with siderophore or iron-chelating function were identified: the catecholate siderophore bacillibactin (Region 9; 100% similarity to BGC0002695), the hydroxamate siderophore schizokinen (Region 4; 60% to BGC0002683), and the iron-chelating cyclic dipeptide pulcherriminic acid (contig 2 region; 66% to BGC0002103).

The detection of a lanthipeptide cluster with 100% similarity to lichenicidin VK21 (BGC0000525) suggests the presence of an antimicrobial defense system that may confer a competitive advantage in the polymicrobial hot spring community. Lichenicidin acts by forming pores in target membranes with documented activity against Gram-positive competitors (Dischinger et al., 2009).

*In silico* analyses using tools such as AMRFinderPlus, antiSMASH, and BAGEL4 have enabled the identification of putative antimicrobial resistance genes, BGCs, and bacitracin clusters. However, these predictions have important limitations: they are database-dependent, may miss novel or highly divergent genes, and do not by themselves confirm gene expression, metabolite production, or phenotypic antibiotic resistance. In particular, the detection of a putative AMR gene or secondary metabolite cluster does not guarantee that the gene or cluster is functional, expressed under the tested conditions, or sufficient to confer resistance or produce a bioactive compound. Experimental validation is therefore necessary to establish a direct relationship between predicted determinants and their biological activity. In addition, the resistance and biosynthetic potential of *B. licheniformis* strains can vary, and other regulatory, physiological, or ecological factors may influence the observed phenotype.

## Conclusion

In conclusion, we isolated, sequenced, and *de novo* assembled the whole genomes of two *B. licheniformis* strains from hot springs in Yellowstone National Park. Our study provides significant insights into the genomic landscape of *B. licheniformis* by identifying the AMR genes and characterizing multiple biosynthetic gene clusters. A comprehensive exploration of biosynthetic gene clusters (BGCs) in *B. licheniformis* strains offers valuable insights into their genomic landscapes. This enhanced understanding paves the way for the discovery of bioactive compounds with distinct properties. The implications of this research extend across various sectors, including drug discovery, agriculture, and biotechnology, underscoring the critical role of natural products in advancing these fields.

## Data Availability

The datasets presented in this study can be found in online repositories. The names of the repository/repositories and accession number(s) can be found below: https://www.ncbi.nlm.nih.gov/, PRJNA1457743 https://www.ncbi.nlm.nih.gov/, SRR38265527 https://www.ncbi.nlm.nih.gov/, SRR38265528 https://www.ncbi.nlm.nih.gov/genbank/, SAMN57473759 https://www.ncbi.nlm.nih.gov/genbank/, SAMN57473760 https://www.ncbi.nlm.nih.gov/genbank/, JBXTWN000000000 https://www.ncbi.nlm.nih.gov/genbank/, JBXTWO000000000.
